# Higher Number of EBI3 Cells in Mucosal Chronic Hyperplastic Candidiasis May Serve to Regulate IL-17-Producing Cells

**DOI:** 10.3390/jof7070533

**Published:** 2021-06-30

**Authors:** Ailish Williams, Helen Rogers, David Williams, Xiao-Qing Wei, Damian Farnell, Sue Wozniak, Adam Jones

**Affiliations:** 1 School of Dentistry, Cardiff University, Heath Park, Cardiff CF14 4XY, UK; Williamsaj24@cardiff.ac.uk (A.W.); weix1@cardiff.ac.uk (X.-Q.W.); Farnelld@cardiff.ac.uk (D.F.); 2 Bristol Dental School, Lower Maudlin Street, Bristol BS1 3NU, UK; Helen.rogers2@UHBristol.nhs.uk; 3 Dental Hospital, University Hospital of Wales, Heath Park, Cardiff CF14 4XY, UK; sue.wozniak@wales.nhs.uk (S.W.); jonesa108@cardiff.ac.uk (A.J.)

**Keywords:** inflammatory cells, chronic hyperplastic candidosis, immunohistochemistry, cytokines

## Abstract

Previous research into the inflammatory cell infiltrate of chronic hyperplastic candidosis (CHC) determined that the immune response is primarily composed of T cells, the majority of which are T helper (CD4^+^) cells. This present investigation used immunohistochemistry to further delineate the inflammatory cell infiltrate in CHC. Cells profiled were those expressing IL-17A cytokine, EBI3 and IL-12A subunits of the IL-35 cytokine, and FoxP3^+^ cells. Squamous cell papilloma (with *Candida* infection) and oral lichen planus tissues served as comparative controls to understand the local immune responses to *Candida* infection. The results demonstrated that *Candida*-induced inflammation and immune regulation co-exist in the oral mucosa of CHC and that high prevalence of cells expressing the EBI3 cytokine subunit may play an important role in this regulation. This balance between inflammation and immune tolerance toward invading *Candida* in the oral mucosa may be critical in determining progress of infection.

## 1. Introduction

Oral candidoses are a collection of infections of the mouth caused by fungi of the genus *Candida*. These infections occur in many people at some point during their lifetime, and although they are typically superficial, there is an elevated risk of systemic candidoses in severely debilitated patients [1]. This is important, as when they occur, systemic candidoses have a high level of morbidity and mortality [2].

Primary forms of oral candidoses include acute/chronic pseudomembranous candidosis, acute erythematous candidosis, chronic erythematous candidosis, and chronic hyperplastic candidosis (CHC). Oral candidoses have distinct features, relating to infection site, clinical presentation, and associated risk factors [3]. Risk factors typically change the local environment and promote microbial dysbiosis, with a corresponding increase in *Candida* numbers. Risk factors may directly influence competing micro-organisms, such as receipt of broad-spectrum antibiotics, or may affect the host’s immune system. CHC is associated with tobacco smoking [4], high frequency intake of alcohol [5], and has a higher prevalence in middle-aged men [3].

CHC presents as white plaque-like lesions, frequently at the commissures of the mouth, on the buccal mucosa, or on the lateral border of the tongue. The infection is distinct from other forms of oral candidosis, as penetration of the oral mucosa by *Candida albicans* hyphae occurs. In response to this hyphal invasion, there is accompanying inflammatory cell infiltrate and hyperplasia of the infected epithelium [6]. Of particular concern, CHC has been linked to premalignant transformation at the lesion site [7]. The precise role of *Candida* in this process is unclear, with some researchers suggesting involvement of *Candida* in the production of carcinogenic nitrosamines [8] and enhancement of the carcinogenic potential of chemicals from tobacco smoking and alcohol consumption [9]. However, others proposed that CHC is a secondary infection of a pre-existing leukoplakia [10]. Importantly, it was reported that timely administration of antifungal agents can resolve CHC [11].

Given the role of host immunity in controlling oral candidosis, a thorough understanding of the immune processes involved is important. The situation is complex, as differential immune responses can occur depending on the relative prevalence of yeast and hyphal forms of *C. albicans*. Mouse models have been used to assess the nature of innate resident immune cells, including gamma delta T (γδ T) cells and innate lymphoid cells (ILCs), as well as infiltrated adaptive inflammatory CD3^+^/CD4^+^ T cells [12,13]. These studies showed that innate and adaptive immune cells play important roles during *Candida* infection by producing the cytokine IL-17A. IL-17A attracts neutrophils to infection sites, which is followed by promoting macrophage function [14]; therefore, it stimulates fungicidal activity [12]. CD3^+^/CD4^+^ Th17 cells are likely to be the primary sources of IL-17A during mucosal candidosis [15]. An over-production of IL-17A at an infection site can, however, result in inflammatory tissue damage. The novel anti-inflammatory cytokine, IL-35 (consisting of EBI3 and IL-12A protein subunits), is produced at infection sites by M2 macrophages or Foxp3^−^ Treg cells (iTr(35)) and this cytokine counters both IL-17A and Th17 cell activity during infection [16].

Previously, we showed that the T cells in CHC are predominantly T-helper (CD4^+^) cells [17]. Furthermore, our research showed that *C. albicans* induces M2 macrophages to produce IL-35 (EBI3/IL-12A) and suppresses Th1 cells through inhibition of IL-12p70 [18]. This present study aimed to expand upon these findings by detecting IL-17A-producing immune cells in CHC together with the regulatory factors of EBI3, IL-12A, and Foxp3^+^ cells. The profile of these components in CHC was also compared with other oral mucosal conditions, namely squamous cell papilloma (SqP, with *Candida* presence) and lichen planus (LP). These findings further increase our knowledge and understanding of host immune responses in CHC.

## 2. Materials and Methods

Tissue biopsies were acquired from patients attending the University Dental Hospital, Cardiff, for diagnostic purposes. Ethical approval was subsequently obtained (IRAS Project ID 136258, 18 June 2014) and permission granted from the Cardiff and Vale University Health Board R&D (14/DEN/5953) to use the tissues for research. Tissues included chronic hyperplastic candidosis (CHC; *n* = 10), squamous cell papilloma (SqP; *n* = 5, with secondary *Candida* colonisation), and oral lichen planus (LP; *n* = 8, negative for *Candida* and positive control for inflammation). The tissues were blinded to researchers using an alphanumeric code during analysis.

Tissue sections were initially stained with haematoxylin and eosin (H&E) using an automated slide staining system (Linistain, Thermo Scientific, Paisley, UK). Subsequently, immunohistochemical staining was undertaken using primary rabbit antibodies (Abcam, Cambridge, UK) against human IL-12A (p35 subunit; Treg, Th1), IL-17A (Th-17), EBI3 Epstein–Barr virus induced gene 3 (Treg, Th1), and Foxp3 (Forkhead box protein 3; Treg). EnVision™ FLEX Antibody Diluent was used to dilute antibodies as follows: IL-12A (1:2000), IL-17A (1:1000), EBI3 (1:100), and Foxp3 (1:400).

For manual antibody detection, we used the Dako REAL™ EnVision™ Detection System as recommended by the manufacturer. Briefly, paraffin sections were cut at 4 µm and floated onto Dako Flex™ slides, dried in oven for 1 h (60 °C), heated at 97 °C in a Dako PT module chamber with pH target retrieval solution (20 min), then cooled to 65 °C. Sections were washed in Dako Wash Buffer (2×) for 5 min followed by Dako HP block (5 min) and washed. Sections were incubated as follows with washing between stages: primary antibodies (20 min), rabbit link reagent (15 min), Flex/HRP polymer (20 min), DAB working solution (10 min), and counterstaining with Dako Flex Haematoxylin (5 min). Sections were then rinsed in distilled water, dehydrated, and overlaid with a coverslip. Negative controls were exclusion of the primary antibody. Images of stained sections were obtained and digitised using an Aperio system (Leica Microsystems Ltd., Milton Keynes, UK).

Histopathological analysis of the stained tissue sections was undertaken using QuPath [19]. Image type brightfield H-DAB was selected and digital ‘demarcation’ was conducted for the epithelium and corium. Three representative areas were selected from each region and automated total count of positive stained cells undertaken.

Data were explored using SPSS V26 with results found to be generally right-skewed. The right skew could not be corrected by logarithmic transformation and potential outliers might have occurred. However, due to small sample sizes we could not rule out that these outliers might have been part of the tail end of the right-skewed distribution, and so these data points were retained in the analysis. Robust statistical approaches were therefore used. Differences between groups were investigated via the Kruskal–Wallis (non-parametric) test followed by Dunn’s post hoc test (both via the statistical package, R V3.6.1). Spearman’s correlation coefficient was used to explore the associations between variables (SPSS V26).

## 3. Results

### 3.1. Detection of IL-17A, EBI,3 and IL-12A Cytokine-Producing Cells, and Foxp3^+^ Cells in the Corium and Epithelial Layer of the Oral Mucosa in CHC, SqP, and LP

**Figure 1:**
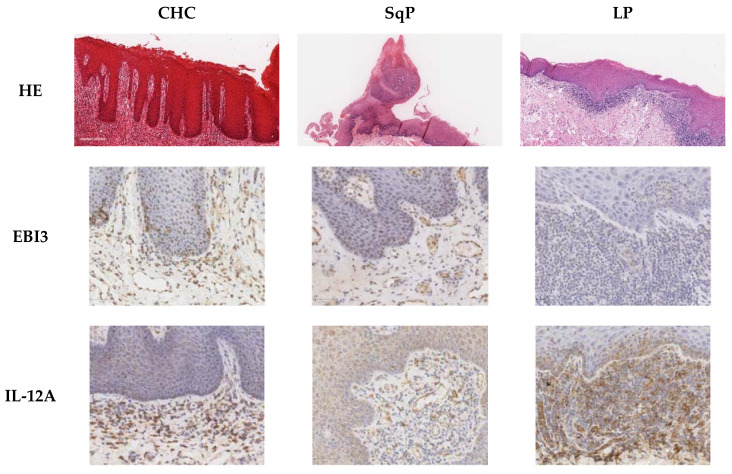

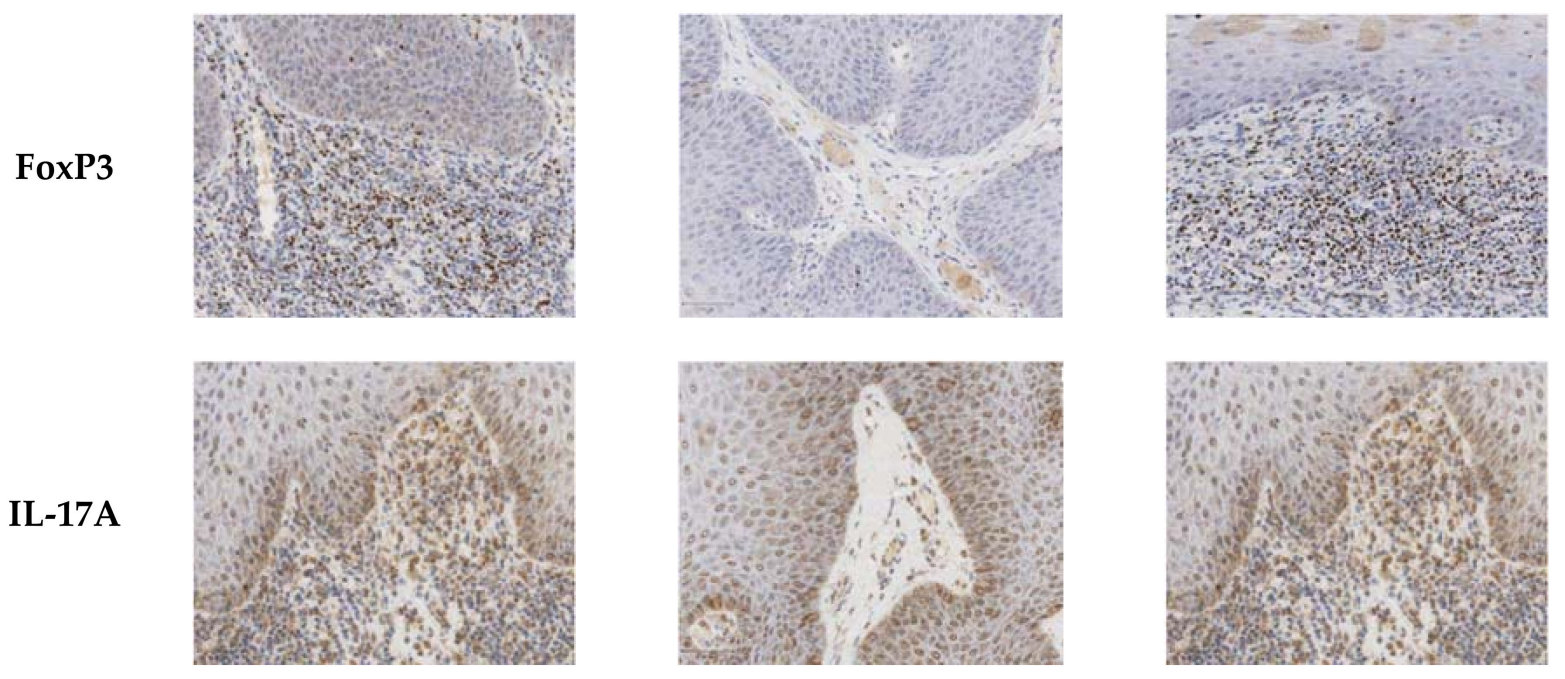
Composite image of haematoxylin and eosin (HE) and immunohistochemical staining of sections of chronic hyperplastic candidosis (CHC), squamous papilloma (SqP), and oral lichen planus (LP). Immunohistochemical staining was for EBI3- Epstein–Barr virus induced gene 3 (Treg, Th1), IL-12A (p35 subunit; Treg, Th1), Foxp3 (Forkhead box protein 3; Treg), and IL-17A (Th-17). Original magnification 100×, except for first row of HE stained sections (7× CHC, 4× SqP, and 7× LP).

The pro-inflammatory cytokine, IL-17A, and its regulatory factors of EBI3 and IL-12A (which combine to form the anti-inflammatory cytokine IL-35) are likely to be highly relevant factors in combating *Candida* infection [20]. Foxp3 is exclusively produced by Treg cells, which perform an important immune-suppressing role during infection [21]. Using immunohistochemistry (Figure 1), the number of cells producing and expressing IL-17A, EBI3, IL-12A, and Foxp3 was determined in both the corium (lamina propria or connective tissue) and the epithelial layers of CHC, SqP, and LP.

### 3.2. Comparison of IL-17A-Producing Cell Numbers in CHC, LP, and SqP

**Figure 2:**
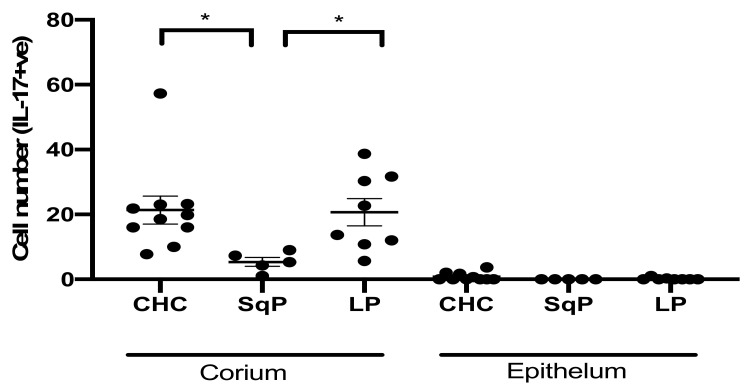
Comparison of IL-17A producing cell numbers in tissue sections of CHC, SqP, and LP. Data are expressed as mean ± standard error. The highest numbers of IL-17A producing cells were detected in the corium (connective tissue) of CHC and LP. SqP (all with *Candida* co-infection) had significantly lower numbers of IL-17A^+^ cells (Dunn’s post-hoc test: *p* = 0.002 for CHC vs. SqP and *p* = 0.003 for LP vs. SqP). Few cells were positive for IL-17A in the epithelial layer of CHC, whereas almost no IL-17A-producing cells were detected in SqP and LP. * *p* < 0.05.

IL-17A-producing cells were detected at the highest levels in CHC and oral lichen planus (Figure 2) and were most prevalent in the corium. Importantly, we previously showed that the epithelial layer of these same tissues contains high numbers of CD4^+^ T cells [17]. This result suggests that either the CD4^+^ Th17 cells had lost the ability to produce IL-17A when infiltrating the epithelial layer, or the majority IL-17A-producing cells were innate immune cells, such as γδT and ILCs. Interestingly, SqP (with secondary *Candida* infection) had significantly lower numbers of IL-17A expressing cells (Dunn’s post hoc test: *p* = 0.044 for CHC vs. SqP), which might suggest higher immune tolerance to *Candida* in SqP.

### 3.3. Comparison of Foxp3^+^ Cell Numbers in CHC, LP, and SqP

**Figure 3:**
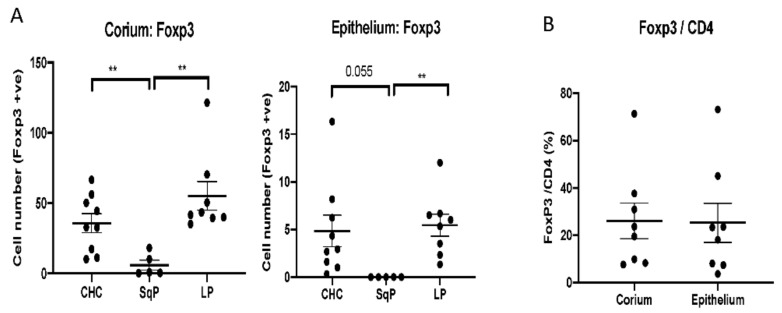
Comparison of Foxp3^+^ cell numbers in tissue sections of CHC, SqP and LP. Data are expressed as mean ± standard error. (**A**) Higher numbers of Foxp3^+^ cells were detected in CHC and LP tissues compared to SqP. Foxp3^+^ cells were detected in both the corium (connective tissue) and epithelium of CHC and LP, but not in SqP. (**B**) As a proportion of previously determined CD4^+^ cells, Foxp3^+^ cells were lower in the epithelium compared to the corium by a factor 2.0 for CHC (Mann−Whitney test: *p* = 0.340) and a factor of 4.3 for LP (Mann−Whitney test: *p* = 0.009). ** *p* < 0.01.

Foxp3-expressing cells are almost exclusively regulatory T (Treg) cells. As with Th17 cells, Foxp3^+^ Treg cells are also dependent on TGFβ1 for their expansion [22,23]. Significantly higher numbers of Foxp3^+^ cells were detected in both CHC and oral lichen planus compared to SqP (Dunn’s post hoc test, connective tissue: ρ = 0.011 for CHC vs. SqP and *p* < 0.001 for LP vs. SqP; Dunn’s post hoc test, epithelium: *p* = 0.003 for CHC vs. SqP and ρ < 0.001 for LP vs. SqP). Significantly higher numbers of Foxp3^+^ cells were again found in the corium compared to the epithelial layer (Figure 3) (Mann–Whitney test: CHC, *p* < 0.001; SqP, *p* = 0.032; LP, *p* < 0.001). Based on the numbers of CD4^+^ T-cells in CHC tissue sections, Treg cells comprised a lower proportion in the epithelium compared to the corium. Although Treg cells play an important role in immune regulation and tolerance, SqP with *Candida* infection did not exhibit high numbers of Foxp3^+^ cells, which may indicate that the immune tolerance mechanism in SqP was different from that of the other tissues. Higher Th17 and Foxp3^+^ Treg cell numbers may be due to TGFβ1-dependent cell development mechanisms.

### 3.4. Comparison of EBI3- and IL-12A-Producing Cell Numbers in CHC, LP, and SqP

**Figure 4:**
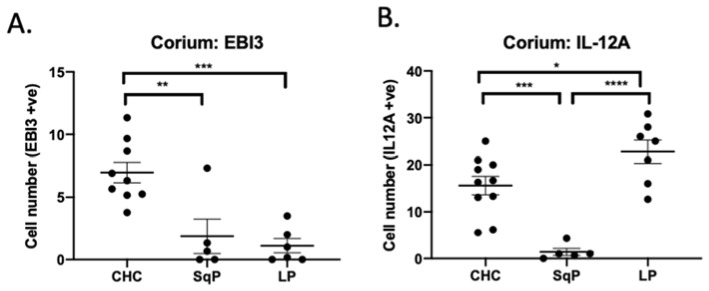
Comparison of EBI3^+^- and IL-12A-producing cell numbers in tissue sections of CHC, SqP, and LP. Data are expressed as mean ± standard error. (**A**) Cells expressing EBI3 were evident in CHC but were in lower numbers in SqP and LP. Significantly higher numbers of EBI3^+^ cells were detected in the corium of CHC compared to SqP and LP. No EBI3^+^ cells were found in the epithelium of any of these conditions. (**B**) Numbers of IL-12A-producing cells were not significantly different in the corium of CHC and LP (Dunn’s post hoc test: *p* = 0.481). * *p* < 0.05; ** *p* < 0.01; *** *p* ≤ 0.001; **** *p* ≤ 0.0001.

Along with IL-12A, EBI3 is a protein subunit of the immune-suppressing cytokine-IL-35. In mice and humans, IL-35 induces immune tolerance by suppressing Th17 and other innate IL-17A-producing cells [16]. The number of EBI3-producing cells in CHC in the corium was much higher than for both SqP and LP (Figure 4) (Dunn’s post hoc test: *p* = 0.007 for CHC vs. SqP and *p* = 0.001 for CHC vs. LP). Higher numbers of EBI3 -positive cells were found in the corium of CHC, which might explain the lower numbers of IL-17A-producing cells previously encountered compared to LP. Numbers of IL-12A^+^ cells were significantly higher in the corium of LP compared to CHC (Dunn’s post hoc test: *p* = 0.481). Since IL-12A also combines with another protein subunit (IL-12p70) for Th1 cell induction, this might explain the previously reported high CD8^+^ T cell numbers detected in LP [15]. CHC and LP had similar levels of Foxp3^+^ Treg cells, but there were higher numbers of EBI3 cells in CHC. This result suggests that immune regulation in CHC might be predominantly performed by IL-35-producing cells (iTr(35)) or IL-35-producing macrophages, and not by Foxp3^+^ Treg cells. This result also agrees with our previous published data showing that *C. albicans* induces higher EBI3 for IL-35 production in M2 macrophages [18]. Higher IL-12A-producing cells in LP might contribute to IL-12p70 production. IL-12p70 is a key cytokine for inducing Th1 responses to further stimulate CD8^+^ T cell expansion. This result also agrees with our previous research of higher CD8^+^ T cells being present in LP than in CHC [17].

### 3.5. Correlation between EBI3- and IL-17A-Producing Cell Numbers in Tissue Sections

A correlation analysis was performed to elucidate the relationship between Foxp3^+^ Treg cell numbers and EBI3 production in the regulation of IL-17A-producing cells in CHC (Table 1). Foxp3^+^ cell numbers had a lower positive association with numbers of IL-17A-producing cells in CHC (Spearman’s correlation coefficient, ρ = 0.08) compared to LP (ρ = 0.524). However, a relatively higher correlation coefficient (ρ = 0.55) was evident between IL-17A and EBI3 in CHC. This indicates that the induction of EBI3, rather than Foxp3^+^ Treg cells, may control IL-17A over-production in CHC. This was previously observed in *C. albicans* induced M2 macrophages for EBI3 production [18]. However, in LP, the host immune system may use Foxp3^+^ Treg to suppress IL-17A-mediated inflammation. These results were, however, not significant, possibly due to the small sample sizes. Further data need to be generated in future studies to increase sample sizes to substantiate this observation.

## 4. Discussion

The features of *Candida* infection differ depending on body location. The most frequent forms of candidosis occur on dermal and mucosal surfaces, particularly those of the oral and vaginal cavities. *Candida* invasion of the body and subsequent systemic infection is comparatively rare; however, when it occurs, it has a high mortality rate, with most cases involving immunocompromised patients.

This study used immunohistochemistry to profile cells expressing the cytokine IL-17A, the cytokine subunits of EBI3 and IL-12A, and the gene transcription protein Foxp3. This was undertaken to enhance our understanding of the host immune response involved in the control of *Candida* at mucosal sites. Tissue sections included those of CHC, and comparative control tissue sections of SqP (with *Candida* infection) and oral lichen planus. The latter is a condition that exhibits autoimmune features with higher CD8^+^ T cell and may initially be triggered by viral infection. Given the possible association of CHC with malignant transformation at the lesion site [7], it is highly important that there is a thorough understanding of host immune responses to the infection. Such information would be beneficial in the diagnosis and prognosis of CHC.

IL-17A is a key cytokine produced by host innate and adaptive immune cells in mucosal tissues and considered important in protection against *Candida* invasion [15,24,25]. EBI3 and IL-12A are protein subunits involved in the formation of members of the IL-12 family of cytokines including IL-35 and IL-12p70. EBI3 and IL-12A combine to form the cytokine IL-35. IL-35 has a strong immune-suppressing role on IL-17A production by both innate and adaptive lymphocytes including γδT cells, innate lymphoid cells (ILCs), and CD4^+^ Th17 cells. IL-12p70 is a critical cytokine for Th1 response in promoting CD8^+^ cytotoxic T cells.

IL-17A-producing cells are thought to be instrumental in the control of *Candida* invasion through IL-17A-mediated recruitment of neutrophils and macrophages. In addition to the important role of IL-17A in defence, IL-17A is also significant in inflammation and autoimmune disorders. Over-production of IL-17A can lead to extensive inflammatory damage to the mucosal barrier, which can, in turn, facilitate *Candida* tissue invasion and dissemination. To counter IL-17A over-production, the host develops an immune regulation response through Foxp3^+^ Treg cells and production of EBI3 and IL-12A protein subunits (combining to form the anti-inflammatory IL-35 cytokine). We showed that IL-35 is a strong immune regulatory cytokine in the control of IL-17A production by Th17 cells at mucosal sites [16,20]. This delicate balance of fungicidal and inflammatory responses is likely to be key in the appropriate restriction of *Candida* infection. Examining inflammatory and anti-inflammatory cytokine production including Foxp3^+^ Treg cells in mucosal sections of CHC will add to our understanding of the host mechanism of defence in this important form of oral candidosis.

Previously, we also showed that *Candida* induces EBI3 production by mouse macrophages, which then participate in immune tolerance by suppressing IL-12p70 and promoting IL-35 production [18]. In this present study, a higher number of EBI3-expressing cells was detected in CHC, which was not evident in SqP and LP tissues. This extrapolates our previous finding that *Candida* may induce immune tolerance in CHC via stimulation of EBI3 production [18].

Immunohistochemistry could not co-detect the necessary biomarkers to identify the specific cell types involved in cytokine production. However, based on our knowledge of mucosal immune cells, we presume that the IL-17A-producing cells were primarily of three cell types, namely innate γδT, ILCs, and adaptive Th17 cells. EBI3 and IL-12A may be generated by Foxp3^−^ Treg cells, dendritic cells, and macrophages, which we have previously shown to be able to produce IL-35 [16,18,20]. We showed that the numbers of IL-17A-producing cells positively correlated with EBI3^+^ cell numbers in the corium of CHC. In contrast, the numbers of cells producing EBI3 and IL-17A showed a greatly reduced correlation with Foxp3^+^ expressing cells (which were almost exclusively Treg cells). However, sample sizes were small and so the results of the correlation analysis should be treated with caution. This finding may indicate that either Foxp3^+^ Treg cells were not present in sufficient numbers to control over-production of IL-17, or that the host response to the *Candida* infection employed EBI3 as the primary suppressing mechanism for IL-17A. Of note, IL-17^+^ cell numbers had a higher association with Foxp3^+^ cells in the autoimmune condition of LP. Interestingly, although SqP tissues also had secondary *Candida* infection, the numbers of all immune cytokine-producing cells and Foxp3 cells were noticeably lower. This result may indicate a severe immune tolerance existing at the SqP tissue site, which may be developed through different mechanism(s) of immune regulation.

The findings of this study suggest that both inflammation and immune regulation co-exist within the mucosal tissues of CHC, and that EBI3 may play an important role in the immune regulation of this *Candida* infection. Whereas Foxp3^+^ Treg cells were detected in CHC, the number of Foxp3^+^ Treg cells demonstrated marginal correlations with IL-17A producing cells. This may indicate that Foxp3^+^ Treg cells, which also suppress Th1 and Th2 immune responses, may either not coexist or are involved in sequential and timely control of inflammation. Further mechanistic studies are warranted to address the roles of EBI3 and IL-35 in *Candida* infection in humans using higher patient numbers and appropriate laboratory experiments.

## Figures and Tables

**Table 1:** Spearman’s correlation coefficient, ρ, between IL-17A and EBI3, or Foxp3 in CHC and LP.

**IL-17A in Lamina Propria of CHC**	**EBI3**	**Foxp3**
ρ	0.55	0.08
*p* =	0.13	0.85
*n*	9	9
**IL-17A in Lamina Propria of Lichen Planus**	**EBI3**	**Foxp3**
ρ	0.116	0.524
*p* =	0.827	0.183
*n*	6	8

Coefficients of magnitude ρ (rho) > 0.5 considered moderate.

## Data Availability

The data presented in this manuscript are available upon reasonable request from the corresponding author.
